# Finite Element Analysis Modelling of Negative Pressure Wound Therapy Drapes

**DOI:** 10.7759/cureus.33412

**Published:** 2023-01-05

**Authors:** Amy K McNulty, Robert Wilkes, Jason Bjork, Michael Turnbull, James Sieracki

**Affiliations:** 1 Medical Solutions Division, 3M, St. Paul, USA; 2 Medical Solutions Division, 3M, San Antonio, USA; 3 Corporate Research Materials Lab, 3M, St. Paul, USA

**Keywords:** vacuum assisted closure drape, principal strain, vacuum-assisted closure, finite-element model, negative-pressure wound therapy

## Abstract

Negative pressure wound therapy (NPWT) drape removal from the skin may be painful for patients and inadvertently cause skin damage during the length of therapy. Most NPWT drapes utilize an acrylate adhesive to achieve the seal. To improve the experience associated with NPWT drape removal, a novel hybrid drape was developed. This drape is composed of areas of acrylate adhesive and areas of silicone adhesive. To more fully understand how the removal of the hybrid drape versus the acrylate drape affects the skin, drape removal models were developed to assess the differences in strain profiles for acrylate versus hybrid NPWT drapes using finite element analysis (FEA) to measure the strain and deformation that occurs at the tissue interface with the NPWT drape. The FEA modeling showed that the maximum principal strain associated with the removal of the acrylate drape was 47.3%, whereas the maximum principal strain associated with the removal of the hybrid drape was 21.5%. The average peel force associated with the acrylate drape was 66.1 gf/in, while the peel force for the hybrid drape was 112.5 gf/in. NPWT drape removal may, in certain instances, be related to pain and periwound skin injury. The hybrid drape tested may provide clinicians with an option for NPWT that is gentler for the skin.

## Introduction

The epidermis is the topmost layer of the skin, which is composed of keratinocytes in various stages of differentiation. After the age of 20, the thickness of the epidermis decreases by about 6.4% per decade [1]. The deepest layer in the epidermis is the stratum basale. The keratinocytes of this layer reside on the dermoepidermal junction (DEJ), which separates the epidermis from the dermis. The DEJ structurally supports the keratinocytes, and its anchoring filaments and fibers help to adhere the epidermis to the upper layer of the dermis, termed the papillary dermis. It is these anchoring structures that most likely increase the resistance of the skin to injury from friction. The DEJ also has a distinct pattern of projections termed "rete ridges" or "rete pegs." These projections extend from the epidermis into the papillary dermis, imparting strength and shear injury resistance to the dermal-epidermal interface [2].

Studies on human basement membranes in various tissues have suggested that with advanced age, the strength of the DEJ decreases [3,4]. As the skin ages, the DEJ flattens, which can lead to an approximate 35% decrease in contact between the dermis and epidermis [4-7]. With age, the fibrils that anchor the various layers of the epidermis to the dermis become stiffer, and skin elasticity decreases due to a fraying of the elastic fibers [8]. These age-related changes in the skin and the DEJ increase the skin’s fragility, making it more prone to trauma and shear injuries. Consensus documents have cited medical adhesives as contributing factors to skin injuries, including skin tears [9-11].

Negative pressure wound therapy (NPWT) drape removal from the skin may be painful for patients and inadvertently cause skin damage during the length of therapy [12]. Most NPWT drapes utilize an acrylate adhesive to achieve the seal. To improve the experience associated with NPWT drape removal, a novel hybrid drape was developed. This drape is composed of areas of acrylate adhesive and areas of silicone adhesive. Silicone adhesives are known to be gentler on the skin than most acrylate adhesives.

There is no clear consensus in the literature as to the strain levels that are damaging to the skin. To date, no in vivo method exists that can measure stresses and strains imparted to the skin with adhesive product removal. Instead, finite element analysis (FEA) modeling must be used. Through the use of mathematical equations and computational modeling, FEA develops numerical values for stresses and strains (tissue deformation) that can develop in the skin in response to loading.

The current study assessed the differences in strain profiles for acrylate (TAC) versus hybrid NPWT drapes using an FEA to measure the strain and deformation that occurs at the tissue interface with the NPWT drapes. This article was previously presented as a meeting abstract at the 2022 Symposium on Advanced Wound Care Spring/Wound Healing Society meeting on April 8, 2022.

## Materials and methods

Peel Force Measurements

Skin mimic substrates were comprised of a 0.05-mm-thick top layer of ethylene vinyl acetate (EVA) membrane film (3M™ CoTran™ Film 9702, 3M Company, St. Paul, MN), a 5-mm viscoelastic silicone layer consisting of Dragon Skin™ FX-Pro™ platinum silicone rubber Part A and Part B (1:1), 1.25 parts Slacker™ silicone tackifier (Smooth-On Incorporated, Macungie, PA), and a 0.5-mm rigid polyethylene terephthalate glycol (PETG) base and one-inch-wide drape samples were applied to the EVA film side of the silicone skin mimics using a 4.5-pound roller with one forward and one backward motion. The skin mimic’s PETG surface was then adhered to an aluminum plate with double-sided tape. This assembly was then clamped into an automated linear indexing system which matched lateral movement with vertical displacement from a TA.XT Plus texture analyzer (Stable Micro Systems, Surrey, UK). The coordinated movement of these components allowed for a fixed focal point of a 90o peel front of the tape from the skin mimic. Peel forces were recorded with the texture analyzer while the adhesive products were removed at 12 inches/minute.

Physical Characterizations

For adhesive thickness testing, a 1-inch-diameter circular area was used to measure total product thickness using a CDI thickness gauge (Chicago Dial Indicator, Des Plaines, IL). A microscope (Dino-Lite Edge, Torrance, CA) was used to image three points per 1-inch sample to determine the ratio of backing to the adhesive of the product. Three samples per product type were used to generate the resultant product and adhesive thicknesses.

The overall modulus of each product was measured using a ZwickRoell Z2.5 (Zwick/Roell, Kennesaw, GA). Product samples were slit to a 10-mm width and loaded into the jaws of the tensile tester. Each product was tested to failure at a crosshead speed of 10 inches per minute.

For measuring the modulus of adhesives, the adhesive is first removed from the drape backing and placed between Dynamic Mechanical Analyzer (DMA) device plates (TA Instruments, New Castle, DE). A moderate normal weight of approximately 50 g is applied to the sample to ensure constant contact between the adhesive and the plates. The material model is built from a calibration data set obtained through dynamic mechanical and thermal analysis (DMTA). In the DMTA test, the material is subjected to small oscillatory shear strains at frequencies between 0.01 and 100 Hz while stepping through a range of temperatures. Due to the time-temperature superposition (TTS) principle, an equivalency can be made between temperature and frequency shifts. A master curve is then constructed, which defines the modulus versus frequency curve at a single reference temperature over a frequency range (~10-5 -1010 Hz).

FEA Test Method

A two-dimensional plane strain model of a 180° peel test was constructed using Abaqus/CAE 2020 finite element analysis software run on an HP Z Book laptop with an Intel Core I7, 9th generation, 6th core processor (HP, Palo Alto, CA). The direction of the tape pull was aligned with the X-axis. The Y-axis was normal to the skin surface. This model of human skin is similar to the in vitro model and consists of a 10-mm-thick base layer of Dragon Skin™ FX Pro silicone rubber (dermal layer), a 0.023-mm-thick layer of acrylate adhesive, and a 0.051-mm-thick layer of polyurethane film (3M™ CoTran™ Film 9701, 3M Company, St. Paul, MN) (epidermal layer). The material properties were determined from the previously described mechanical tests. Material damping (alpha = 100,000, beta = 0) was added to the Dragon Skin™ material to absorb kinetic energy. The FEA model was 15 mm long and 5 mm thick. The non-linear model was meshed with 9,901 rectangular elements at 0.1-mm lateral resolution per element for the models shown in Figures 2, 3. The vertical mesh resolution was smaller and varied with depth and material. The bottom and left boundaries of the skin model were fixed. The top and right boundaries were free within the two-dimensional plane.

The drape portion of the model consisted of two layers for the traditional acrylate drape (3M™ VAC® Drape, 3M Company, St. Paul, MN) and five layers for the hybrid drape (3M™ Dermatac™ Drape, 3M Company, St. Paul, MN), using drape component thicknesses shown in Table 1. The thermoplastic polyurethane (TPU) film’s Young’s moduli were inferred from the drape mechanical tests. The viscoelastic adhesive’s storage and loss moduli at 1, 10, and 100 Hz were determined from the DMTA test data.

The model was run as a dynamic simulation lasting 2.1 seconds. Initiation of the 180° peel was done by displacing the upper left corner node of the tape pull tab 0.5 mm in both positive X and Y directions while rotating the node +1.5 radians to fold the tape back on itself over a time span of 100 ms. The pull tab was then displaced in the positive X direction for 10 mm during a time span of 2 s (5 mm/sec) to simulate the peel test. The force required to pull the tape versus time was recorded and plotted. The time point where the force most closely approximates the mean peel force from the skin mimic test served as a snapshot of the skin peel dynamics. Heat map plots of the models at this time point were used to illustrate the deformation and strain magnitudes.

## Results

Table 1 shows the physical thicknesses of the various drape components and the drape construction's overall modulus. The hybrid drape is approximately four-fold thicker than the traditional acrylate drape. This thickness increase is mostly due to the presence of the silicone adhesive layer, which is over 0.2 mm thick in the hybrid drape. The hybrid drape had the stiffest overall drape construction, with a modulus of 47.2 MPa.

**Table 1:** Drape component thicknesses and modulus TPU: Thermoplastic polyurethane

Traditional drape		Hybrid drape	
Acrylate adhesive (mm)	0.061	Silicone adhesive (mm)	0.2032
TPU backing (mm)	0.025	TPU film 1 (mm)	0.0305
Modulus (MPa)	10	Acrylate adhesive (mm)	0.0508
		Acrylate adhesive (mm)	0.0406
		TPU film 2 (mm)	0.028
		Modulus (MPa)	47.2

Table 2 shows the storage modulus was lowest for the silicone adhesives used in the hybrid drape at all frequencies tested. Between 1 and 100 Hz, it was between 21.6- and 32.9-fold less than the storage modulus of the acrylate used in the traditional acrylate drape. For the hybrid drape, the silicone adhesive had a storage modulus between 17.8% and 11.4% of the acrylate storage modulus used in the same hybrid drape. Table 3 shows G"/G’ values over the frequency sweeps for the various adhesives used in the drapes. The traditional acrylate has the highest glass transition value, which would correspond to a rapid increase in glass transition just below room temperature, with the hybrid drape acrylate adhesive showing a similar trend and the second-highest glass transition value. The silicone adhesive shows a much lower glass transition value, as evidenced by its relatively flat G"/G’ (tan delta) values.

**Table 2:** DMA viscoelasticity data for adhesive drapes DMA: Dynamic mechanical analyzer

	Storage modulus (MPa)			Loss modulus (MPa)		
	1 Hz	10 Hz	100 Hz	1 Hz	10 Hz	100 Hz
Acrylate, traditional drape	0.1704	0.3677	1.05	0.1013	0.3253	1.4
Acrylate, hybrid drape	0.0443	0.0983	0.281	0.0281	0.0821	0.274
Silicone, hybrid drape	0.0079	0.0152	0.0319	0.0036	0.0086	0.019

**Table 3:** G”/G’ values for adhesive drapes

	1Hz	10Hz	100Hz
Acrylate, traditional drape	0.5945	0.8847	1.3333
Acrylate, hybrid drape	0.6343	0.8352	0.9751
Silicone, hybrid drape	0.4557	0.5658	0.5956

**Figure 1:**
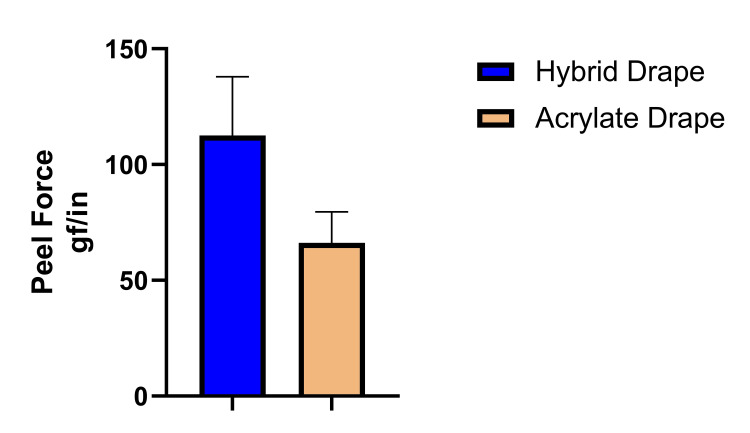
Peel force values for the negative pressure wound drapes. One-inch strips of drapes were removed from the skin mimic at 12 in/min and a peel angle of 90 degrees.

The peel force of the drapes from the skin mimic ranged from 66.2 ± 13.3 gf/in for the traditional acrylate drape to 112.5 ± 25.4 gf/in for the hybrid drape (Figure 1). The differences in the means did not reach statistical significance (p>0.05).

**Figure 2:**
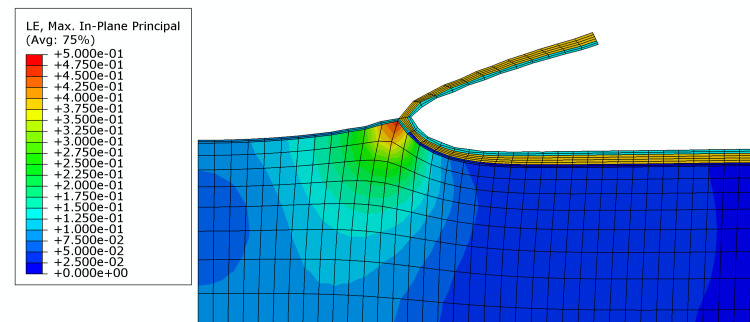
FEA model for traditional acrylate drape. Colors represent the principal in-plane expansive strain. The maximum principal strain is 47.3%, and it occurs in a small area directly under the peel front. FEA: Finite element analysis

**Figure 3:**
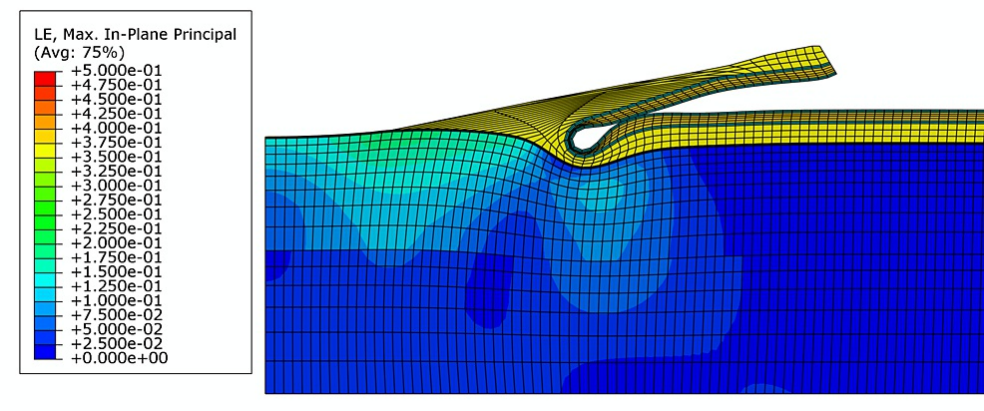
FEA model for hybrid drape. Colors represent the principal in-plane expansive strain. The maximum principal strain is 21.5%, and it occurs in an area under and behind the peel front. FEA: Finite element analysis

The maximum principal strain associated with drape removal was the lowest for the hybrid drape (21.5%). This was less than half the maximum principal strain associated with the removal of the traditional acrylate drape (TAC) (47.3%) (Figures 2-3; Table 4). Maximum principal strain contour plots for each drape are presented in Figures 2-3, which illustrate areas of high strain at the leading edge of the peel on the traditional acrylate drape.

**Table 4:** Maximum (expansive) and minimum (compressive) strain values for all drapes

	Traditional acrylate Drape	Hybrid drape
Maximum principal strain	47.3%	21.5%
Minimum principal strain	-20.5	-26.1%

## Discussion

The modeling in the current study represents the strain (deformational) forces experienced by the skin when NPWT drapes are removed. During removal, layers of the stratum corneum are removed with adhesive tapes, and different tape constructions may remove or separate these epidermal layers at different rates [13]. Medical adhesives can also cause mechanical separation of the epidermis and the dermis. These types of skin damage have been termed medical adhesive-related skin injuries (MARSI), which can range from skin stripping to skin tears. MARSI is an important consideration in high-risk patients such as those with fragile skin and is believed to affect approximately 27% of individuals with postoperative wounds [11,14].

Silicone tapes have been shown to remove fewer epidermal cells [15]. This class of adhesives is softer than acrylate adhesives. This fact was clearly illustrated in the silicone storage modulus of the hybrid drape shown in Table 4. The storage modulus for the silicone adhesive used in the hybrid drape was a minimum of 21.6-fold less than the acrylate from the traditional NPWT drape, indicating that the silicone adhesive used in this drape was much less stiff. A softer adhesive is more easily deformed, which could produce a broader peel front as the tape is removed. Therefore, the forces experienced at the peel front may be distributed over a larger area as the adhesive stretches, leading to a lower peak strain at the peel front. The reverse may be true for overall construction stiffness, which is mainly influenced by the backing. It was shown that Young’s modulus was highest for the hybrid drape. A stiffer drape construction would be associated with a larger peel front bend radius, which would also provide a larger area for the distribution of stress through the adhesive into the skin. The modeling results presented herein indicate that the hybrid drape imparted over 50% less maximum principal strain to the skin upon removal than the TAC drape. These maximum strain values are due to expansive deformation of the skin, analogous to pulling or stretching of the tissue. Clinically, large, expansive strains could be more damaging to the skin in patients more predisposed to MARSI.

The G"/G’ value of a pressure-sensitive adhesive (PSA) provides information about glass transition. Effective PSAs are neither too brittle nor too viscous. Since test frequency and temperature are inversely related, the G"/G’ values shown in Table 3 indicate that the TAC drape has a glass transition at a higher temperature than the adhesives used in the hybrid drape. This could indicate that the adhesives used in the hybrid drape (and especially the silicone) retain their PSA functionality over a broader range. Further, for a cohesively strong PSA, G"/G’ (tan delta) values between 0.6 and 0.5 are ideal. The silicone in the hybrid drape with a G"/G’ between 0.46 and 0.6 may therefore have good cohesive strength and remove cleanly. Further testing needs to be conducted to verify this finding.

Limitations

While this study has revealed important biomechanics associated with the removal of NPWT drapes, it is important to discuss some of the limitations in the modeling approach. Firstly, as with all FEA tissue models, homogeneity of the tissue is assumed, whereas actual tissue has multiscale variations in these properties. The net result of this is that it is likely that our strains may be overpredicted to some extent. The relative comparisons between the different drapes are still valuable, and the results provide important insights. The skin was modeled using average physical values from the literature. Future studies could consider sensitivity analysis based on the effects of these varying parameters.

## Conclusions

When using medical adhesive products such as NPWT drapes, it is important to choose the best product for the intended job that provides the least potential damage to the skin. For NPWT, it is important that the drape be able to maintain a seal. The data provided showed that the hybrid drapes had adhesion (peel) values to the skin surrogate that were at least equivalent to the traditional acrylate drape. The p-value associated with the peel force of the hybrid drape, although not reaching statistical significance, was highly suggestive that the peel value would be statistically higher than the traditional acrylate drape if a higher number of replicates were conducted. The FEA model indicated that removal of the hybrid drape imparted less than half as much maximum principal strain to the skin as the traditional acrylate drape. While the traditional acrylate drape is very effective at maintaining a seal and is thus a very good option for NPWT, the hybrid drape presents an alternative option that may be beneficial for patients, especially those with more frail skin or skin predisposed to skin damage such as skin tears.
